# The Level of hs-CRP in Coronary Artery Ectasia and Its Response to Statin and Angiotensin-Converting Enzyme Inhibitor Treatment

**DOI:** 10.1155/2007/89649

**Published:** 2006-12-27

**Authors:** Yilmaz Ozbay, Mehmet Akbulut, Mehmet Balin, Hidayet Kayancicek, Adil Baydas, Hasan Korkmaz

**Affiliations:** Department of Cardiology, School of Medicine, Firat University, 23200 Elazığ, Turkey

## Abstract

*Background/Aim*. Coronary artery ectasia (CAE) was thought of as a variant of atherosclerosis. C-reactive protein (CRP) which is among the most sensitive markers of systemic inflammation, and elevation of systemic and local levels of this inflammatory marker which has been associated with an increased risk for cardiovascular disease in the obstructive coronary artery disease (O-CAD) are well known, but little was known in CAE. The anti-inflammatory effects of statins and the effect of angiotensin-converting enzyme (ACE) inhibitors on endothelial dysfunction are well established in atherosclerosis. The aim of the present study was to investigate CRP level and its response to statin and ACE inhibitor treatment in CAE. *Materials and method*. We measured serum hs-CRP level in 40 CAE (26 males, mean age: 56.32 ± 9 years) and 41 O-CAD (34 males, mean age: 57.19 ± 10 years) patients referred for elective coronary angiography at baseline and after 3-month statin and ACE inhibitor treatment. *Results*. Plasma hs-CRP levels were significantly higher in CAE group than O-CAD group at baseline (2.68 ± 66 mg/L versus 1, 64 ± 64, resp., *P* < .0001). Plasma hs-CRP levels significantly decreased from baseline 3 months later in the CE (from 2.68±0.66 mg/L to 1.2±0.53 mg/L, *P* < .0001) as well as in the O-CAD group (from 1.64±0.64 mg/L to 1.01±0.56 mg/L, *P* < .001). *Conclusion*. We think that hs-CRP measurement may be a good prognostic value in CAE patients as in stenotic ones. Further placebo-controlled studies are needed to evaluate the clinical significance of this decrease in hs-CRP.

## 1. INTRODUCTION

Coronary artery ectasia (CAE) was defined as localized or diffuse dilation of ≥ 1.5 times normal adjacent segments of vessels [1]. It is estimated that 50% coronary artery ectasia is related to atherosclerosis, whereas 20%–30% of cases may be due to congenital anomalies [2–4], and CAE was found in the range of 1.2–4.9% in different series [1, 5]. Markis et al. revealed that the underlying histological changes were identical to those found in atherosclerotic lesions (diffuse hyalinization, intimal, and medial damages) [6]. However, it is not known clear why some patients with obstructive coronary artery disease develop CAE, whereas most do not. CRP is a sensitive marker of systemic inflammation [7]. The elevation of systemic and local levels of this inflammatory marker has been associated with an increased risk for cardiovascular disease [8]. Although the CRP in obstructive coronary artery disease (O-CAD) is well known, limited data are available in CAE and there are conflicting results [9, 10].

Accordingly, in this study we compared the plasma hs-CRP levels in patients with isolated CAE and O-CAD and their responses to statin (simvastatin 40 mg/day) and angiotensin-converting enzyme (ACE) inhibitor (perindopril 8 mg/day) treatment.

## 2. MATERIALS AND METHODS

### 2.1. Patients

This study was conducted between January 2005 and December 2005. The study population was selected from 2174 consecutive patients who underwent coronary angiography in our clinic. The first group consisted of 40 patients (CAE group, 26 males, mean age: 56.32 ± 9 years) with isolated CAE without significant stenosis. These patients were selected from individuals referred for coronary angiography due to the presence of typical angina and/or abnormal noninvasive test results suggesting myocardial ischemia. A total 53 (2.43%) CAE patients were found and 13 of them were excluded from the study due to ACE inhibitor or statin therapy. A well-matched control group (41 patients, O-CAD group, 34 males, mean age: 57.19 ± 10 years) was selected from patients referred for elective coronary angiography due to presence of typical angina and/or abnormal noninvasive test results suggesting myocardial ischemia and subsequently was found to have significant coronary artery stenosis, and any revascularization was not planned before first blood sample was obtained. The local ethics committee approved the study protocol. Written informed consent was obtained prior to enrolment.

Those patients with collagen vascular disease, active inflammation, acute coronary syndrome history, under ACE inhibitor and statin treatment were not included in the study.

### 2.2. Coronary angiograms and immunoassay

The coronary angiograms were performed by Judkin's technique in multiple projections without the use of nitroglycerine. Ultravist 370 (Schering AG, Germany) was used as the contrast agent in all patients. Two experienced invasive cardiologists who were blinded to patients' informations and laboratory results evaluated the angiographies of patients. CAE was considered when a vessel diameter was ≥ 1.5 times that of adjacent normal segment. If no adjacent normal segment could be identified, the mean diameters of the normal coronary segments in stenotic patients served as normal values. A significant stenosis was defined as a ≥ 50% loss of the diameter near to adjacent normal segment.

Blood samples of all individual were taken 24 hours after coronary angiography from an antecubital vein. Blood samples were centrifuged immediately at 250 g for 10 minutes and stored at +4°C until assay. The hs-CRP assayments were done by Dade Behring BN II (Germany).

After blood sample was collected, simvastatin (40 mg daily) and perindopril (8 mg daily) were added to the therapy of all individuals. A second blood sample was collected from all individuals by the same method 3 months later.

### 2.3. Statistical analyses

All data were analyzed by SPSS software (Statistical Package for the Social Sciences), version 12.0 for Windows. Data were presented as mean ± standard deviation unless noted as different. Comparisons between groups were analyzed using the nonparametric Mann-Whitney *U* test. The relationship between the number of vessel involvements and hs-CRP was determined by linear regression analyses. Basal and 3-month hs-CRP levels with respect to number of involved vessels were determined by Spearman's correlation. *P* value of < .05 was considered statistically significant.

## 3. RESULTS

**Figure 1:**
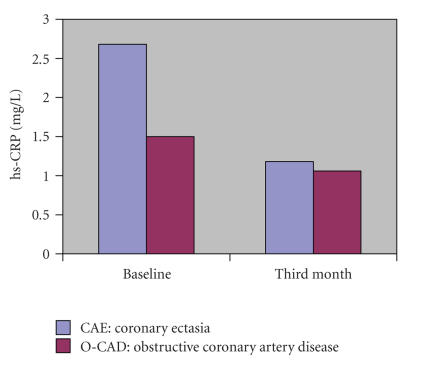
The hs-CRP levels in both groups at baseline and third month.

Baseline clinical characteristics of the two groups were similar (Table 1). The angiographic characteristics of CAE were presented in Table 2. Serum hs-CRP levels were significantly higher in CAE group than O-CAD group at baseline (2.68 ± 66 mg/L versus 1, 64 ± 64, resp., *P* < .0001) (Figure 1). Those patients with multivessel involvement of CAE or O-CAD had higher serum hs-CRP levels than single involvement (*P* < .001, 95%Cl; 0.285–0.625, *R* ^2^ = 0.263). There was no significant correlation between average diameter of CAE and hs-CRP (*r* = 0.244, *P* = .129), and a weak correlation was observed between average ectasia diameter and hypertension (HT) (*r* = 0.300, *P* = .06). However there was a positive correlation between hs-CRP and length of ectasia (*r* = 0.332, *P* = .037). A positive correlation was observed between smoking and hs-CRP in O-CAD and CAE (*r* = 0.430, *P* = .005; *r* = 0.587, *P* < .0001, resp.). When hs-CRP was analyzed with respect to age, a positive correlation was found in O-CAD group and no correlation was found in CAE group (*r* = 0.401, *P* = .09; *r* = 0.173, *P* = .287). A positive correlation was observed between hs-CRP and LDL-C in both CAE and O-CAD groups (*r* = 354, *P* = .001; *r* = 0.280, *P* = .011, resp.). There was a positive correlation between hs-CRP and diabetes mellitus (DM) (*r* = 0.473, *P* = .002) but no correlation with HT in O-CAD group (*r* = 0.087, *P* = .590). However, there was no correlation between hs-CRP and DM and HT in CAE group (*r* = 0.681, *P* = .067; *r* = 0.673, *P* = .069).

Serum hs-CRP levels significantly decreased from baseline 3 months later in the CAE (from 2.68 ± 0.66 mg/L to 1.2 ± 0.53 mg/L, *P* < .0001) as well as in the O-CAD group (from 1.64 ± 0.64 mg/L to 1.01 ± 0.56 mg/L, *P* < .001). However, the quantitative and proportional reduction was significantly higher in the CAE group than the O-CAD group (68% versus 45% (*P* < .001)). Most significant decreases in hs-CRP were observed in patients with higher baseline hs-CRP levels (*P* < .1, *R* ^2^ = 0.617) (Table 3). The hs-CRP levels became similar after 3-month treatment (1.2 ± 0.53 mg/L in CAE versus 1.01 ± 0.56 mg/L in O-CAD group, *P* > .05) (Figure 1).

## 4. DISCUSSION

CAE was found in the range of 1.2%–4.9% in different series [5, 6]. It causes adverse coronary events like vasospasm, dissection, and thrombosis [5]. There is still controversy about the pathogenetic mechanism that underlies this entity. It is estimated that 50% of CAE is related to atherosclerosis and others are related to congenital or inflammatory disease like Kawasaki syndrome. Extensive structural damage was observed in different layers of vessel especially in the tunica media and intima in histological examinations [5, 11].

Inflammatory process begins from the earliest phase of atherosclerosis, formation of fatty streak, involving the leukocytes infiltration and link between plaque formation and acute plaque rupture, leading to acute coronary syndromes [12]. The acute-phase reactant, CRP, a simple marker of inflammation, has now emerged as a major cardiovascular risk factor [13]. High-sensitive CRP (hs-CRP) was shown to be an independent risk factor for MI, stroke, sudden death, and peripheral arterial disease in different prospective epidemiological studies and it can be decreased by statins independent of the LDL-level reductions [14–16]. It was shown that angiotensin II induces inflammatory changes in human vascular smooth muscle cells; and in animal models of atherosclerosis, this inflammation was suppressed by ACE inhibitors [17, 18]. Tsikouris et al. reported that quinapril (higher tissue penetration than enalapril) had stronger effect on hs-CRP reduction than enalapril following myocardial infarction [19]. And in the EUROPA study, beneficial effect of perindopril on future cardiovascular events in stable angina patients was shown [20].

Although large body of information about the inflammatory process underlying the O-CAD is present, limited data are available about the role of inflammation in the pathogenesis of CAE.

In this study, we found significantly higher levels of hs-CRP in CAE when compared to O-CAD. Turhan et al. found similar results with our findings, whereas Finkelstein found that hs-CRP levels were similar in both CAE and O-CAD [9, 10]. We think that this conflicting result may be due to the study population. Most of the patients in Finkelstein study were using statins or ACE inhibitors at the beginning of the study (82.3%, 52.9%, resp.) and these agents might suppress the inflammatory process. Besides these findings, extensive inflammation including medial and adventitial cell infiltrations was observed in postmortem examination of aortic aneurysm, and higher serum CRP levels were observed in asymptomatic aortic aneurysm persons [21, 22]. We suggest that this extensive inflammation is responsible for higher hs-CRP levels in CAE. We have shown that this higher hs-CRP level responded to statin and ACE inhibitor treatment dramatically and again significant reduction was observed in O-CAD group and hs-CRP levels meet the same level at the end of 3-month treatment. We believe that the suppression of the inflammation may prevent adverse coronary events in CAE.

In multivariable analysis, hs-CRP was found significantly correlated with smoking, LDL-C, and a weak correlation between HT and ectasia diameter, but no correlation was observed between hs-CRP and age and DM in CAE group, whereas a significant correlation was found with respect to age, DM, smoking, LDL-C in O-CAD group. In previous studies, smoking was represented as a risk factor for CAE [23, 24]. We think that smoking seems to induce adverse events in CAE beside its effect on pathogenesis of CAE. The finding of any correlation between hs-CRP and age in CAE group is correlated with the previous report of CAE seen in younger-age group [25]. In a large- scale study; Androulakis et al. reported an inverse relation between CAE and DM [26]. In our study, we choose patients with similar risk factor profiles in both groups, so we did not have chance to reach such a conclusion, but in our CAE group no correlation was observed between hs-CRP and DM supporting this finding.

In conclusion, we found that hs-CRP levels were significantly higher in CAE patients than O-CAD. And this higher inflammatory process responded to statin and ACE inhibitor therapy dramatically at the end of 3-month therapy. We think that hs-CRP measurement may be a good prognostic value in CAE patients as in stenotic ones. Further placebo-controlled studies are needed to evaluate the clinical significance of this decrease in hs-CRP.

## Figures and Tables

**Table 1:** Baseline clinical characteristics of the patients. CAE denotes coronary ectasia and O-CAD denotes obstructive coronary artery disease.

	CAE	O-CAD
	(*n* = 40)	(*n* = 41)

Age (mean±SD) (years)	57.19±10	56.32±9
Sex (men)	32	34
Diabetes mellitus	21	26
Hypertension	18	13
LDL-cholesterol (mg/dL)	115	121
Smoking	17	12
Family history	25	24
Aspirin	40 (100%)	41 (100%)
Nitrate	31 (77.5%)	28 (68.29%)
Beta blockers	35 (87.5%)	36 (87.8%)

**Table 2:** Angiographic findings of ectasias.

Distribution of ectasia	

Left anterior descending artery	20 (50%)
Circumflex	23 (57%)
Right coronary artery	24 (60%)

Number of involved vessels	

1	17 (42,5%)
2	18 (45%)
3	5 (12.5%)

**Table 3:** hs-CRP Levels (mg/L) and number of vessel involvements. CAE denotes coronary ectasia and O-CAD denotes obstructive coronary artery disease.

Number of vessel involvements	CAE		O-CAD	
	(*n* = 40)		(*n* = 41)	

	Basal	3 months	Basal	3 months

Single	2.20 (17)	1.16	1.03 (17)	0.95

Multiple	3.03 (23)	1.24	1.93 (24)	1.04
